# Modified Heat-Stable Toxins (hSTa) of Enterotoxigenic *Escherichia coli* Lose Toxicity but Display Antigenicity after Being Genetically Fused to Heat-Labile Toxoid LT(R192G)

**DOI:** 10.3390/toxins3091146

**Published:** 2011-09-15

**Authors:** Mei Liu, Chengxian Zhang, Kristy Mateo, James P. Nataro, Donald C. Robertson, Weiping Zhang

**Affiliations:** 1 Veterinary & Biomedical Sciences Department, The Center for Infectious Disease Research & Vaccinology, South Dakota State University, Brookings, SD 57007, USA; Email: mei.liu@sdstate.edu (M.L.); chengxian.zhang@sdstate.edu (C.Z.); kristy.mateo@sdstate.edu (K.M.); 2 Department of Pediatrics, University of Virginia School of Medicine, Charlottesville, VA 22908, USA; Email: JPN2R@hscmail.mcc.virginia.edu; 3 Department of Diagnostic Medicine/Pathobiology, Kansas State University, Manhattan, KS 66506, USA; Email: droberts@vet.k-state.edu

**Keywords:** STa toxin, ETEC, toxoid, LT-STa toxoid fusion, vaccine, diarrhea

## Abstract

Enterotoxigenic *Escherichia coli* (ETEC) strains are a major cause of diarrhea in humans and animals. Heat-stable (STa) and heat-labile (LT) enterotoxins produced by ETEC disrupt fluid homeostasis in host small intestinal epithelial cells and cause fluid and electrolyte hyper-secretion that leads to diarrhea. ETEC strains producing STa or LT are sufficiently virulent to cause diarrhea, therefore STa and LT antigens must be included in ETEC vaccines. However, potent toxicity and poor immunogenicity (of STa) prevent them from being directly applied as vaccine components. While LT toxoids, especially LT(R192G), being used in vaccine development, STa toxoids have not been included. A recent study (IAI, 78:316-325) demonstrated porcine-type STa toxoids [pSTa(P12F) and pSTa(A13Q)] elicited protective anti-STa antibodies after being fused to a porcine-type LT toxoid [pLT(R192G)]. In this study, we substituted the 8th, 9th, 16th, or the 17th amino acid of a human-type STa (hSTa) and generated 28 modified STa peptides. We tested each STa peptide for toxicity and structure integrity, and found nearly all modified STa proteins showed structure alteration and toxicity reduction. Based on structure similarity and toxic activity, three modified STa peptides: STa(E8A), STa(T16Q) and STa(G17S), were selected to construct LT_192_-STa_-toxoid_ fusions. Constructed fusions were used to immunize mice, and immunized mice developed anti-STa antibodies. Results from this study provide useful information in developing toxoid vaccines against ETEC diarrhea.

## 1. Introduction

A recent systematic survey indicated that diarrhea kills up to 2 million children under the age of five, more than AIDS, malaria and measles combined [1,2]. Enterotoxigenic *Escherichia coli* (ETEC) strains that colonize host small intestines and produce one or two enterotoxins are the major bacterial cause of children’s diarrhea, and are responsible for approximately 200 million episodes of diarrhea and 380,000 deaths annually [3]. In addition, ETEC strains are also the most common cause of travellers’ diarrhea [4,5]. Although experimental vaccines currently under development show promising, no broadly effective vaccines are available to protect humans and animals against ETEC diarrhea. The key virulence factors of ETEC in diarrhea are colonization factors and enterotoxins [6,7,8,9,10,11,12]. Colonization factors mediate initial attachment of ETEC bacteria to host small intestinal enterocytes and subsequent colonization. Enterotoxins known as heat-stable (STa) and heat-labile (LT) toxins disrupt fluid homeostasis and cause fluid and electrolyte hyper-secretion through activation of adenyl cyclase (by LT) or guanylate cyclase (by STa) in small intestinal epithelial cells that leads to diarrhea [13,14].

LT and STa toxins, in addition to colonization factors are proven the virulence determinants in ETEC diarrhea. LT is a typical 1A:5B toxin that consists of one enzymatic A subunit (25.5 KDa) and five identical GM1-binding B subunits (12 KDa), and is strongly immunogenic. In contrast, STa associated with human diarrhea (hSTa; STa used in following context is referred as hSTa) is a small peptide of only 19 amino acids (2 KDa) and is poorly immunogenic [10]. Epidemiological and clinical studies showed that one half of human diarrheal cases associated with ETEC are caused by strains that produce STa toxin only, a quarter express LT only, and another quarter produce both toxins [15,16]. Human volunteers and animal challenge studies demonstrated that an enterotoxigenic *E. coli* strain expressing either STa or LT toxin is sufficiently virulent to cause diarrhea [10,11,12,17]. Therefore, both STa and LT toxins must be targeted in vaccine development against ETEC diarrhea [18,19,20,21]. Indeed, experimental vaccine studies indicated that anti-LT immunity alone is not sufficiently effective in protection against ETEC. Vaccine candidates carrying LT antigens provided protection against only LT-producing ETEC strains, but not against ETEC strains expressing STa toxin [22,23]. It becomes commonly acknowledged that anti-STa immunity must also be induced by vaccines for effective protection against ETEC diarrhea. 

The potent toxicity of LT and STa, however, must be attenuated, and the poor immunogenicity of STa must be enhanced before LT and STa can be used as safe and effective vaccine antigens. LT toxoids, especially LT(R192G) that has the Arg^192^ substituted with Gly, have toxicity much reduced but LT immunogenicity (and adjuvanticity) maintained and were used as antigens in ETEC vaccine development [24,25,26]. This LT(R192G) has also been used as an adjuvant to enhance immunogenicity of otherwise poorly immunogenic antigens [27,28,29,30,31,32]. In contrast, although early studies demonstrated a few modified STa peptides including STa_12_, STa_13_ and STa_14_ showed toxicity reduction [33,34], STa toxoids have not been included in ETEC vaccine development until recently [35,36]. Recent studies showed that porcine-type STa toxoids, pSTa(P12F) and pSTa(A13Q) which are the analogues of human-type STa_13_ and STa_14_, had anti-STa immunogenicity enhanced and elicited protective anti-STa antibodies after being genetically fused to toxoid LT(R192G) [35] or *E. coli* K88ac fimbriae [36]. Candidacy of other STa toxoids in vaccine application, however, has not been evaluated. In addition, studies showed that different STa toxoids exhibited differences in toxicity reduction [34] and likely structure alteration [35]. Further studies to examine additional modified STa peptides for correlation between anti-STa immunogenicity (when are carried by a carrier protein) and toxicity reduction or structure integrity will provide instructive information to select STa toxoids in developing ETEC toxoid vaccines.

In this study, we generated various modified STa peptides by substituting the 8th, 9th, 16th and 17th amino acids of STa toxin. The 8th, 9th, 16th and 17th residues located inside the STa toxic domain which consists of a peptide from Cys^6^ to Cys^18^ and constitutes the minimal structure for fully biological activity of the STa toxin [37]. As the 6 cysteines that form three disulfide bonds, Cys^6^ to Cys^11^, Cys^7^ to Cys^15^, and Cys^10^ to Cys^18^, are essential to maintain the STa three-dimensional conformation, and STa_12_, STa_13_, and STa_14_ had already been characterized, the 8th, 9th, 16th and 17th are the only residues left inside the toxicity domain to be studied for their role(s) in STa enterotoxicity and structure integrity. We substituted each of these four amino acids with a residue of different characterization, such as molecular weight, accessible surface area and polarity, and examined each modified STa protein for changes in structure and toxicity. Furthermore, we selected three modified STa peptides based on their structure similarity and toxic activity to construct LT_R192G_-STa_-toxoid_ fusions and examined the fusions for anti-STa antigenicity.

## 2. Materials and Methods

### 2.1. Bacterial Strains and Plasmids

ETEC strain *E. coli* H10407 (O78:H11) was used to isolate the STa gene (*estA*), and *E. coli* BL21 (GE Healthcare, Piscataway, NJ, USA) was used as the host strain in this study. Vector pUC19 (Promega, Madison, WI, USA) was used to clone and express the wildtype and mutated STa genes, and expression vector pET28α (Invitrogen, Carlsbad, CA, USA) was used to express LT and STa toxoid fusions. BL21 strain expressing native STa was used as the positive control, whereas BL21 with vector pUC19 as the negative control. All strains were cultured in LB medium or 4AA minimum medium supplemented with kanamycin (30 µg/mL) or ampicillin (50 µg/mL) accordingly (Table 1).

**Table 1:** *Escherichia coli* strains and plasmids used in this study.

Strains	Relevant properties	Plasmid	Reference
H10407	ETEC prototype strain	LT^+^STa^+^	ATCC #35401
BL21	*E. coliB F^−^*, *omp*T, *hsd*S (r_B_^−^, m_B_^−^), *gal*, *dcm.*		GE Healthcare
8964	LT_R192G_-STa_E8A_ construct, BL21/pLT_R192G_-STa_E8A_	pLT_R192G_-STa_E8A_/pET28α	this study
8968	LT_R192G_-STa_T16Q_ construct, BL21/pLT_R192G_-STa_T16Q_	pLT_R192G_-STa_T16Q_/pET28α	this study
8971	LT_R192G_-STa construct, BL21/pLT_R192G_-STa	pLT_R192G_-STa/pET28α	this study
8975	LT_R192G_-STa_G17S_ construct, BL21/pLT_R192G_-STa_G17S_	pLT_R192G_-STa_G17S_/pET28α	this study
8836	STa recombinant, BL21/pSTa	pSTa/pUC19	this study
8930	negative control, BL21	pUC19	this study

### 2.2. Cloning and Mutation of *estA* Gene

The STa gene (*estA*) was PCR amplified from genomic DNA of ETEC strain H10407 (ATCC #35401) with designed primers hSTapUCHindIII-F (5'-gcgcaaagcttctgattttgat-3'; underlined is a HindIII site) and hSTapUCBamHI-R (5'-agccacggcggatccaaatataaaggg-3'; underlined is a BamHI site) in a PCR as described previously [35]. PCR amplified products were separated in 1.5% agarose gel (FMC Bioproducts, Rockland, MA, USA) and extracted using a QIAquick gel extraction kit (QIAGEN, Valencia, CA, USA). Purified PCR products and plasmid pUC19 were digested with HindIII and BamHI restriction enzymes (New England Biolab, Ipswitch, MA, USA). Digested *estA* gene products and vectors were gel electrophoresed and purified, and ligated with T4 DNA Ligase (New England BioLab). Two microliters of ligation products were used to transform *E. coli* BL21 competent cells under a standard electroporation procedure [38]. Antibiotic resistant colonies were PCR screened first, followed by DNA sequencing to ensure that the cloned *estA* gene stayed in the correct reading frame.

**Figure 1:**
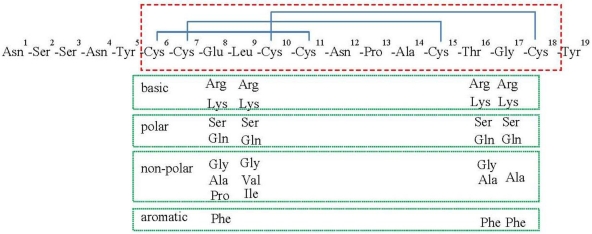
Illustration of a mature STa toxin peptide and substitutions. Amino acid residues in the red-colored dashed box are of STa toxicity domain, and three disulfide bonds are indicated in blue-colored solid lines. Boxes in green-colored solid line indicated amino acid residues used in substitution.

To substitute the 8^th^, 9^th^, 16^th^ and the 17^th^ amino acids of the STa toxin, we designed specific PCR primers to mutate nucleotides encoding these four residues (supplementary Table 1). A list of codons and substitutions were illustrated in Figure 1. We designed primers to substitute the Glu^8^ with Pro, Ala, Phe, Ser, Gly, Arg, Lys or Gln; the Leu^9^ with Val, Ile, Gln, Lys, Arg, Gly or Ser; the Thr^16^ with Ala, Phe, Gln, Lys, Arg, Gly or Ser; and the Gly^17^ with Ala, Lys, Gln, Arg, Ser or Phe (Figure 1). The *eltAB* genes coding LT toxin were PCR amplified from genomic DNA of H10407 and cloned in pUC19 vector, and were mutated with LT_192_-F and LT_192_-R PCR primers [26], for toxoid LT(R192G).

### 2.3. STa Competitive ELISA to Detect STa Proteins Expressed by the Recombinant and Mutant Strains

Reactivity to anti-native STa antiserum (D. Robertson laboratory) was measured to assess structure integrity among the modified STa peptides. Culture growth of the STa recombinant and mutant strains was used in STa competitive ELISA as described previously [35,39]. Briefly, each strain was cultured in LB overnight, and grown culture was measured with optical density (OD). An equivalent amount of cells from each strain was sub-cultured in 4 AA medium at a start OD value of 0.05. After 8 h growth, culture was measured for OD again. Supernatant from an equivalent amount of cells was used in ELISA.

STa ovalbumin-conjugates (1.25 ng; D. Robertson laboratory) were coated at each well in a STa ELISA plate overnight at 37 °C. The coated plate was blocked with 2.5% casein blocking buffer (2.5% casein in 0.3 N NaOH, pH 7.0) at 37 °C for 1 h. Seventy-five microliters of culture supernatant from each strain (in triplicate) and 75 µL of anti-native STa rabbit antiserum (1:10,000 dilution; D. Robertson laboratory) were mixed and added to each well, followed by an incubation at 37 °C for 2 h on a shaker (180 rpm). After three washes, plates were blotted to dry, incubated with horseradish peroxidase (HRP)-conjugated goat anti-rabbit immunoglobulin (IgG, 1:10,000 dilution) (Sigma, St. Louis, MO, USA) at 37 °C for 1 h. OD was measured at a wavelength of 405 nm after 20 min of incubation in peroxidase substrate (KPL, Gaithersburg, MA, USA).

Reactivity to anti-STa antiserum from each mutated STa peptide was measured and compared to that of the recombinant STa to assess protein structure similarity, with an assumption that a modified STa peptide will have similar reactivity to anti-STa antiserum as the recombinant STa peptide if this modified STa peptide maintains similar structure of the recombinant STa. With the reactivity of the recombinant STa peptide to anti-STa antiserum was set as 100% and that of the negative control to antiserum as 0%, reactivity of each modified STa peptide to the anti-STa antiserum was calculated and converted to structural similarity to the native STa.

### 2.4. Intracellular Cyclic GMP ELISA to Detect Toxicity of STa Peptides

Toxicity of the recombinant and mutated STa peptides was determined for stimulating intracellular cyclic GMP in T-84 cells (ATCC #CCL-248) with a direct cyclic GMP enzyme immunoassay kit (Correlate^TM^ EIA, Assay Designs, MI, USA), as described previously [35]. Briefly, 1 × 10^5^ T-84 cells were seeded and cultured in the Dulbecco’s modified Eagle medium (DMEM/F12; GIBCO/Invitrogen, Grand Island, NY, USA). After removal of culture medium, 75 µL overnight-grown supernatant (in 4AA medium, of equivalent amount of cells) from each strain (in duplicate) was added to each well of a 24-well plate, and incubated for 2 h. Cells were lysed with 100 µL 0.1 M HCl and then were neutralized with 0.1 M NaOH. One hundred microliter lyses supernatant was mixed with the reagents (conjugates and antibodies) supplied with the kit, and was added to each well of the supplied EIA plate. After incubation on a shaker (500 rpm) for 2 h at room temperature, plates were washed and blotting dried. Reactivity with supplied pNpp substrate solution (OD value) was measured at 405 nm after 20 min development.

### 2.5. Porcine Ligated Gut Loop Assays to Detect Biological Activity of Expressed STa Proteins

STa proteins expressed by the recombinant and mutant strains were also examined for biological activity in porcine ligated loop assays as described previously [12,17]. Three 7-day old piglets were deprived of food overnight (with supply of water ad libitum), and were surgically prepared for 24, 24, and 20 loops from ileum and lower jejunum segments. Twenty-eight toxoid mutant strains were randomly divided into 3 groups (10, 10, and 8), and the recombinant strain and the negative control were added to each group. Two × 10^9^ CFUs of overnight-grown culture from each strain were injected into each ligated loop (in duplicate; 1–10, +, −; 1–10, +, −). After 8 h post-inoculation, the length of each loop (cm) and amount of fluid accumulated in each loop (g) were measured. The ratio of accumulated fluid to the loop length (g/cm) was calculated to assess enterotoxic activity for each expressed STa protein. A ratio of greater than 0.1 is typically considered as positive of enterotoxicity.

### 2.6. Construction and Expression of LT_R192G_-STa_E8A_, LT_R192G_-STa_T16Q_, LT_R192G_-STa_G17S_, and LT_R192G_-STa Fusions

Three modified STa peptides, STa(E8A), STa(T16Q) and STa(G17S), were selected for fusion construction. Each STa peptide was genetically fused to the LT(R192G) through an L-linker peptide [24] in a three-step PCR method described previously [26,35]. In addition, the native STa was also included in fusion construction. Each fusion gene was cloned into vector pET28α and expressed in *E. coli* BL21 strain as 6xHis-tagged fusion proteins. As the stop codon of LT(R192G), and the transmembrane signal peptides of the LTR(192G) and each STa peptide were removed, each fusion was constructed as one open reading frame and thus expressed as a single protein. 

Total proteins extracted from each fusion strain were used to examine fusion protein expression in a standard Western blot with anti-CT (Sigma) and anti-STa antiserum (D. Robertson laboratory). Fusion strains were grown in LB medium supplemented with kanamycin (30 µg/mL) at 37 °C overnight. Upon the OD_600_ value reached to 0.5, culture growth was induced by adding 1 mM isopropyl-β-D-thiogalactopyranoside (IPTG) and cultured continually for 4 h. Total proteins were extracted using bacterial protein extraction reagent by following the manufacture’s protocol (B-PER, in phosphate buffer; Pierce, Rockford, IL, USA). Thirty microliters of total protein extracts from each fusion strain were used in a standard sodium dodecyl sulfate-polyacrylamide gel electrophoresis (SDS-PAGE) and immunoblot assay [38]. Transferred membrane blots were blocked with 2% fat-free milk overnight at 4 °C, incubated with rabbit anti-CT (1:3000; Sigma) and anti-STa serum (1:5000; Robertson laboratory), respectively. Fusion proteins on the blots recognized by IRDye conjugated goat anti-rabbit IgG (1:5000; LI-COR, Lincoln, NE, USA) were visualized with a LI-COR Odyssey Infared Gel Imaging system (LI-COR). In addition, fusion proteins were also examined in SDS-PAGE with standard Coomassie blue staining.

### 2.7. Extraction and Refolding of Fusion Proteins, and Mouse Immunization

Fusion proteins extracted in the format of inclusion bodies were denatured and refolded before being used in mouse immunization. Proteins extracted as inclusion bodies were refolded using a protein refolding kit with buffer #5 and #9 according to the manufacture’s instruction (Pierce). Three female adult BALB/c mice (Harlan, Indianapolis, IN, USA) per group were immunized intraperitoneally (IP) with each refolded fusion protein. One hundred microgram of fusion proteins, in an equal volume of Freud’s incomplete adjuvant (Sigma), was injected to each mouse in the group. One booster injection was followed two weeks later. One group of 3 mice without injection was used as the control group. All mice were sacrificed two weeks after the booster injection. Blood samples were collected before and 14 days after each injection, and collected serum samples were stored at −80 °C until use.

Anti-STa antibodies were titrated from serum samples in STa ELISA as described previously [35,36]. Briefly, 1.25 ng STa-ovalbumin conjugates was coated in each well of a 96-well plate at 37°C overnight. Each well was blocked with 2.5% casein blocking buffer, and then incubated with the mouse serum samples (in a binary dilution started at 1:40; in triplicate). Serum samples from the control mice were also included. HRP-conjugated goat anti-mouse IgG (1:4000; Sigma) was used as the secondary antibodies. The cutoff OD values in ELISA were defined as the A_405_ of the background plus 0.4. The dilution that gave OD values above the cutoff was calculated for antibody titers which were expressed at the log_10_ scale.

All animal studies complied with the Animal Welfare Act, followed the *Guide for the Care and Use of Laboratory Animals* [40], and were under the supervision of South Dakota State University’s Institutional Animal Care and Use committee.

### 2.8. Statistical Analysis

Data were analyzed using the mixed procedure (SAS for windows, version 8; SAS Institute, Cary, NC, USA), and Student’s *t*-test was used to compare different treatment groups. Data were presented as means and standard deviations. Calculated *p* values of <0.05 were regarded as significant when treatment groups were compared at two-tailed distribution and two-sample equal variance.

## 3. Results

Twenty-eight mutant strains were constructed in this study, and STa proteins expressed from each mutant strain was examined for toxic activity and structure integrity (Table 2). Three out 28 modified STa peptides, STa(E8A), STa(T16Q) and STa(G17S) which showed less changes at structure, toxic activity, or both, were selected for constructing LT_192_-STa_-toxoid_ fusions. Each fusion was used in mouse immunization for assessing the influence of STa structure and/or toxicity in anti-STa antigenicity. 

**Table 2:** Structure alteration and toxicity of STa expressed by mutant strains. Supernatant of overnight growth (in 4 AA medium) from an equal amount of cells for each strain was used in STa competitive ELISA and cGMP ELISA. Reactivity of modified STa peptides to anti-STa serum was expressed as protein structure similarity by referring to the recombinant STa. Crude overnight culture growth of each mutant strain, the recombinant strain, and the negative control strain was used in porcine ligated gut loop assay.

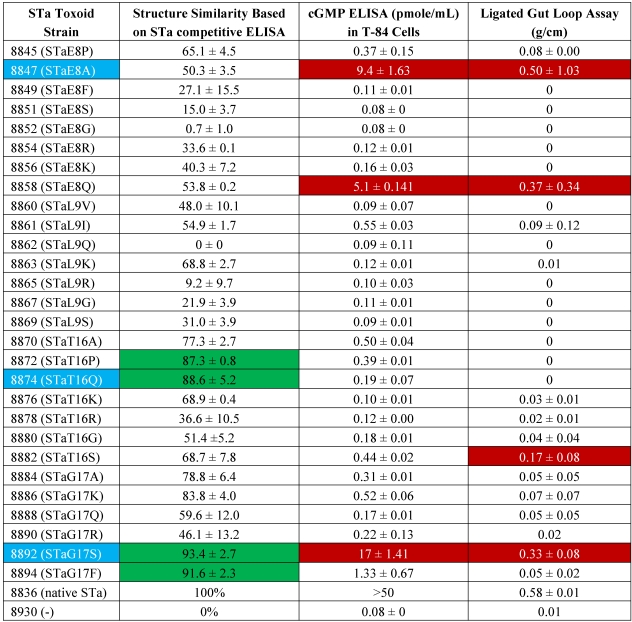

### 3.1. STa Proteins Expressed from Mutant Strains Showed Structure Alteration

The STa genes in the mutant strains and the recombinant strain were verified with DNA sequencing, and expressed STa peptides were examined in STa competitive ELISA with STa-ovalbumin conjugates as the competitive antigens and anti-native STa antiserum as the primary antibodies. ELISA data showed modified STa peptides had a wide range of structure alteration. After being substituted with a Gly at the Glu^8^ or a Gln at the Leu^9^, resultant STa proteins, STa(E8G) and STa(L9Q), no longer reacted to anti-STa serum. That indicated these two modified STa peptides had structure changed significantly. In contrast, the STa(T16P) (87.3 ± 0.8), STa(T16Q) (88.6 ± 5.2), STa(G17F) (91.6 ± 2.3) and STa(G17S) (93.4 ± 2.7) maintained a similar structure as the natural STa (Table 2). Although modified STa peptides showed a wide variation, ranging from 0% to 93.4%, regarding reactivity to anti-STa serum, STa derivatives with mutation at its Glu^8^ or Leu^9^ residue displayed greater structure alteration. The mean reactivity to anti-STa serum for the STa proteins with substitution at the 8^th^ and 9^th^ residues was 35.7% and 33.4%, respectively. In contrast, substitution at the STa_16_ and STa_17_ residues were less sensitive to structure alteration, as STa_16_ and STa_17_ derivative STa proteins showed mean reactivity (to anti-STa antiserum) of 68.4% and 75.6%, respectively.

### 3.2. STa Proteins Expressed from Mutant Strains Showed Toxicity Reduction

STa proteins expressed from all mutant strains showed toxicity reduction. Indeed, STa proteins expressed by most mutant strains showed little or no toxic activity. Only STa(E8A), STa(E8Q), and STa(G17S) showed toxic activity as they stimulated an increase of intracellular cGMP level in T-84 cells. These three mutant strains also had toxic biological activity detected in porcine ligated gut loop assay. Fluid accumulated in the loops incubated with mutant strains expressing STa(E8A), STa(E8Q), and STa(G17S) was measure at 0.50 ± 1.03, 0.37 ± 0.34, and 0.33 ± 0.08 (g/cm), respectively. In addition, the mutant strain expressing STa(T16S) had a positive fluid accumulation (0.17 ± 0.08 g/cm) in ligated gut loops (Table 2).

### 3.3. STa(E8A), STa(T16Q) and STa(G17S) Exhibited Differences in Anti-STa Antigenicity When Were Fused to LT(R192G)

**Figure 2:**
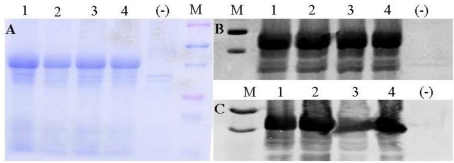
Detection of expressed fusion proteins. Panel A: Total proteins extracted from inclusion body portion were visualized with Coomassie blue staining. The top and bottom purple markers are 75 and 25 KDa, and the two blue markers inside the purple markers are 50 and 37 KDa. Panel B: detection of refolded fusion proteins by anti-CT antiserum. Panel C: detection of refolded fusion proteins by anti-STa antiserum. The two markers in panel B and C are 50 and 37 KDa. Lane 1: LT_R192G_-STa_E8A_; lane 2: LT_R192G_-STa_T16Q_; lane 3: LT_R192G_-STa; lane 4: LT_R192G_-STa_G17S_.

Three STa proteins that retained STa structure or toxic activity were selected to be fused to LT(R192G) for assessing anti-STa immunogenicity. The STa(E8A) which showed toxicity (9.4 ± 1.63 pmole/mL cGMP, 0.50 ± 1.03 g/cm fluid accumulation) but had structure greatly altered (50.3%), STa(T16Q) that showed similar structure to native STa (88.6%) but had toxicity diminished, and STa(G17S) that retained STa structure (93.4%) and much of the toxicity (17 ± 1.4 pmole/mL, 0.33 ± 0.08 g/cm) were individually fused to the LT(R192G), and each fusion protein was used in mouse immunization study. In addition, the wildtype STa was also fused to LT(R192G). All fusion proteins were expressed as inclusion bodies (Figure 2A) which were extracted under denatured condition, but were refolded for natural formation and were verified with anti-CT (Figure 2B) and anti-STa antiserum (Figure 2C).

**Figure 3:**
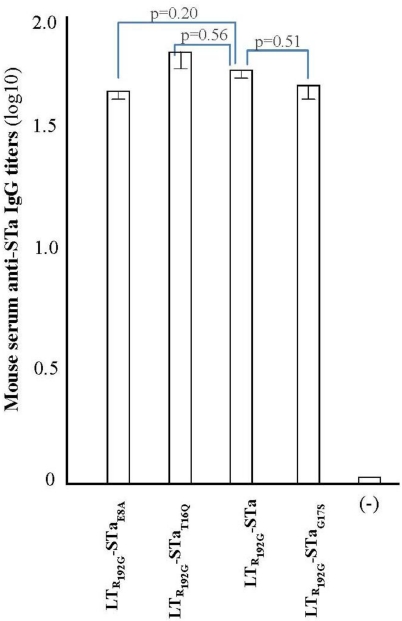
Anti-STa IgG antibody titration from serum samples of mice immunized with LT_R192G_-STa_E8A_, LT_R192G_-STa_T16Q_, LT_R192G_-STa and LT_R192G_-STa_G17S_ fusion proteins. Serum samples from immunized mice (1:40 in dilution) and the control unimmunized mice (1:10 in dilution) were used to titrate anti-STa IgG in a STa ELISA. 1.25 ng STa-ovalbumin conjugates coated at each well as the antigen, and HRP-conjugated goat anti-mouse IgG (1:4000) was used as the secondary antibodies. Boxes and error bars are means and standard deviation.

Antibody titration from serum samples showed that mice immunized with the fusion antigens developed anti-STa antibodies. Anti-STa IgG titers (in log_10_) in serum samples of mice immunized with LT_R192G_-STa_E8A_, LT_R192G_-STa_T16Q_, and LT_R192G_-STa_G17S_ were 1.64 ± 0.04, 1.70 ± 0.07, and 1.65 ± 0.07, respectively. Anti-STa IgG was also detected in mice immunized with LT_R192G_-STa (1.68 ± 0.04). In contrast, no antibodies were detected in the control mice, even when serum samples at a 1:10 dilution were used (Figure 3).

Statistical analyses suggested there were no significant differences at anti-STa IgG antibodies among mice immunized with different fusion antigens. However, anti-STa IgG titers detected in mice immunized with LT_R192G_-STa_T16Q_ and LT_R192G_-STa_G17S_, of which the carried STa had less structure alteration, were closer to that of the group immunized with LT_R192G_-STa (*p* = 0.53, *p* = 0.52). Comparatively, anti-STa IgG antibody titers in the group immunized with LT_R192G_-STa_E8A_ fusion antigen was relatively more different than those of the LT_R192G_-STa immunized group (*p* = 0.20) (Figure 3).

## 4. Discussion

As one of the virulence determinants in ETEC-associated diarrhea, STa needs to be included in ETEC vaccine development. Since STa is toxic and also poorly immunogenic, we need not only to attenuate its potent toxicity but also to facilitate its immunogenicity before we can include STa as a vaccine component [21,35]. STa immunogenicity can be enhanced as an early study showed the native STa became immunogenic when was chemically cross-linked or genetically fused to a high immunogenic carrier protein [21]. However, STa used in these compounds retains toxicity [18,24,41]. The toxicity issue was solved when shorter STa peptides or STa with disulfide bonds disrupted were used for crosslink or genetic fusion. However, resultant compounds failed in inducing neutralizing anti-STa antibodies. Thus, these STa analogues exhibited little value in ETEC vaccine application [42,43]. On the other hand, several non- or low-toxic LT analogues are immunogenic and show ability to induce protective anti-LT immunity [24,25,44,45,46]. But anti-LT immunity alone is not sufficient to protect against ETEC [22,23]. Only vaccines also inducing protective anti-STa immunity could effectively protect against ETEC diarrhea [18,19]. Thus, to identify STa molecules that have low or no toxicity but potentially induce neutralizing anti-STa antibodies, when are cross-linked or fused to a carrier protein, becomes a priority in ETEC vaccine development.

The Asn^12^, Pro^13^ and Ala^14^ of STa were extensively studied for their significance in toxic activity. STa had toxicity reduced or diminished after its Asn^12^, Pro^13^ or Ala^14^ residue being substituted selectively with several different amino acid residues [34]. It was demonstrated that STa showed little or limited toxicity reduction when the Asn^12^ was replaced by a Val or an Ala, or the Pro^13^ by a Val or an Ala, but had a thousand or ten thousand folds reduction in toxicity when the Asn^12^ or Ala^14^ was substituted with an Arg or a Lys residue [34]. It was also revealed STa showed significant variance in toxicity reduction when substitutions occurred at different residues. For an example, STa had toxicity greatly reduced when the Ala^14^ residue was substituted. Data from the current study showed that STa toxicity reduced when the Glu^8^, Leu^9^ Thr^16^, or the Gly^17^ was substituted, and that toxicity reduction varied when either residue was replaced by a different amino acid residue. When the Glu^8^ was replaced by a Phe, Pro, Ser, Gly, Arg or a Lys, STa lost all or nearly all toxic activity. But STa retained much of the toxicity when the same Glu^8^ was substituted by an Ala or a Gln residue, based on the data form porcine ligated gut loop assay. It could suggest that the outcome of toxicity reduction may be associated with basic chemical characteristics of the residues, or its hydrophobic interaction with GG-C [47]. However, this observation is based on only a few substitutions, future systematic studies will be needed for better assessment of any association between basic chemical characters of substitutes and STa toxicity reduction. There is one report that suggested the Gly^17^ resides the inner surface, and with the backbone atoms this Gly^17^ is involved in a side-by-side hydrogen-bonding network (47). Therefore, substitution of Gly^17^ could results in significant changes in STa peptide structure. However, 4 out 6 substitutions {STa(G17S), STa(G17F), STa(G17K), and STa(G17A)} did not cause significant structural alteration in STa as they were well recognized by anti-STa serum. One possible explanation is that we measured structure similarity based on reactivity of STa peptides to the anti-native STa antiserum, and substitutions of Gly^17^ with some amino acid residues may not always necessary result in collapse of the STa topology, thus these STa analogues can still be recognized by the anti-STa antiserum. Clearly, more studies will be needed to further characterize this Gly^17^ residue for significance in STa structure and biological function.

Generally, substitutions of a single amino acid residue could significantly affect a small molecule, such as STa, in structure and biological activity. STa toxin is a peptide of 19 amino acids (18 amino acids in porcine-type STa) including six cysteines forming three disulfide bonds. Thus any changes including a substitution of a single amino acid could disrupt the STa structure and function. Maintenance of the STa structure, especially the epitope topology in fusions or conjugates is believed to be important in eliciting neutralizing anti-STa antibodies (communication with Dr. John Clements). A couple of published papers confirmed that the three disulfide bonds: Cys^6^ to Cys^11^, Cys^7 ^to Cys^15^, orCys^10^ to Cys^18^, are essential for STa to maintain its structure and function [48,49]. Therefore, we did not target these cysteine residues for substitution. In this study, we assessed structure integrity of the modified STa peptides by their reactivity to anti-STa antiserum (anti-native STa antiserum) and found a wide range of variation in structure alteration among these STa analogues. Peptides STa(L9Q) and STa(E8G) showed no or little similarity to the native STa in structure, most likely due to that the Leu^9^ cannot tolerate amino acid substitution without a dramatic drop of STa efficiency [50]. Additionally, both Glu^8^ and Leu^9^ with Cys-Cys adopt a confirmation that tightly interacts with the hydrophobic region of GC-C to achieve maximum potency [51]. That suggests STa(L9Q) and STa(E8G) have the structure or epitope topology changed so substantially that they are no longer recognizable by the anti-STa serum. Even if these two proteins were strongly antigenic, antibodies elicited by these proteins likely would not neutralize native STa toxin. On the other hand, other STa analogues including STa(G17S), STa(G17F), STa(T16Q), STa(T16P) and STa(G17K) showed similar reactivity to anti-STa serum as the recombinant STa. That suggests these STa molecules retained much of the STa native structure or epitope topology. If they become antigenic after being fused or conjugated, the elicited antibodies likely will neutralize STa toxin. While most STa peptides generated in this study showed toxicity reduced or abolished, three peptides STa(E8A), STa(E8Q) and STa(G17S) retained some STa toxicity as each stimulated an increase of cGMP in T-84 cells and also fluid accumulation in ligated porcine gut loops. Differently, toxicity in peptide STa(T16S) was detected only in porcine ligated loop assay but not in cGMP ELISA. However, the toxicity detected in the gut loop assay, 0.17 ± 0.08 g/cm, is relatively lower compared to the other three peptides. That may indicate that STa(T16S) is less toxic and further studies may be needed to characterize this peptide.

The primary goal of this study is to identify STa analogues that retain structure similar to native STa but show no or low toxicity to be used for constructing LT and STa toxoid fusions in ETEC vaccine development. Because of its small size, we expected difficulties to find STa proteins with minimum structure alteration but great toxicity reduction. While most of the modified STa peptides generated in the current study showed changed in both structure and toxicity, a few apparently had structure greatly altered but toxic activity retained {STa(E8A), STa(E8Q)}, or had toxicity lost but with minimum protein structure changed {STa(T16P), STa(T16Q), STa(G17K), STa(G17F)}. Only STa(G17S), however, retained much of the toxicity and the native structure, as it showed over 93% of similarity in reactivity to anti-STa antiserum and nearly half of the toxic activity measured in stimulation of the cGMP level or fluid accumulation in ligated gut loop. To examine whether structure or toxicity plays a more important role in STa antigenicity, we selected STa(E8A) that retained some toxicity but had structure greatly altered, STa(T16Q) that had structure much maintained but toxicity lost, and STa(G17S) showing minimum change in either structure or toxicity to be genetically fused to LT(R192G), and used to immunize mice. Surprisingly, no significant differences were found among these three fusions regarding anti-STa IgG antibodies elicitation. Compared to the fusion of LT(R192G) and the native STa (LT_R192G_-STa), however, STa(T16Q) and STa(G17S) that had structure less altered exhibited less difference in eliciting anti-STa antibodies. That may suggest that STa structure or epitope topology could influence more in anti-STa antigenicity, thus STa toxoids that had minimum structure alteration should be considered primarily as candidates for toxoid fusion construction. However, when porcine-type STa (pSTa) peptides pSTa(P12F) and pSTa(A13Q) were used in toxoid fusion construction, the pLT_R192G_-pSTa_P12F_ stimulated higher titers of anti-STa antibodies, despite pSTa(A13Q) toxoid was structurally more similar to native pSTa [35]. Such contradiction could be caused by a lack of direct comparison to the native pSTa (used in fusion construction) from our previous study. Considering that we only examined a small number of STa analogues, caution needs to be taken to draw conclusions based on observations from this study. Clearly, more studies will be needed to better select STa toxoids for constructing LT and STa toxoid fusions, and perhaps a different animal model, such as a pig model, could help us to examine induced anti-STa antibodies in protection against STa-positive ETEC infection. In addition, further studies to construct a STa toxoid library, not just from the toxicity domain, but also the *N*-terminus, to select more STa peptide representatives for toxoid fusions, and to systematically examine these fusions for anti-STa antigenicity, would help us to identify optimum STa toxoids for ETEC vaccine development. 

## Supplementary Files

Supplementary File 1: DOC-Document (DOC, 39 KB)
