# Supplementary material for: Modified Heat-Stable Toxins (hSTa) of Enterotoxigenic Escherichia coli Lose Toxicity but Display Antigenicity after Being Genetically Fused to Heat-Labile Toxoid LT(R192G)
Source: Toxins (Basel). 2011 Sep 15;3(9):1146–62. doi: 10.3390/toxins3091146 (PMC3202872; doi:10.3390/toxins3091146)
Supplement: Supplementary File 1: — DOC-Document (DOC, 39 KB) [file toxins-03-01146-s001.doc]

**Supplementary Information**

**Mei Liu ^1^, Chengxian Zhang ^1^, Kristy Mateo ^1^, James P. Nataro ^2^, Donald C. Robertson ^3^ and Weiping Zhang ^1^,***

^1^ Veterinary & Biomedical Sciences Department, The Center for Infectious Disease Research & Vaccinology, South Dakota State University, Brookings, SD 57007, USA;
E-Mails: mei.liu@sdstate.edu (M.L.); chengxian.zhang@sdstate.edu (C.Z.); kristy.mateo@sdstate.edu (K.M.)

^2^ Department of Pediatrics, University of Virginia School of Medicine, Charlottesville, VA 22908, USA; E-Mail: JPN2R@hscmail.mcc.virginia.edu

^3^ Department of Diagnostic Medicine/Pathobiology, Kansas State University, Manhattan, KS 66506, USA; E-Mail: droberts@vet.k-state.edu

* Author to whom correspondence should be addressed; E-Mail: weiping.zhang@sdstate.edu;
Tel.: +1-605-688-4317; Fax: +1-605-688-6003.

Received: 29 July 2011; in revised form: 1 September 2011 / Accepted: 6 September 2011 /
Published: 15 September 2011

**Abstract:** Enterotoxigenic *Escherichia coli* (ETEC) strains are a major cause of diarrhea in humans and animals. Heat-stable (STa) and heat-labile (LT) enterotoxins produced by ETEC disrupt fluid homeostasis in host small intestinal epithelial cells and cause fluid and electrolyte hyper-secretion that leads to diarrhea. ETEC strains producing STa or LT are sufficiently virulent to cause diarrhea, therefore STa and LT antigens must be included in ETEC vaccines. However, potent toxicity and poor immunogenicity (of STa) prevent
them from being directly applied as vaccine components. While LT toxoids, especially LT(R192G), being used in vaccine development, STa toxoids have not been included. A recent study (IAI, 78:316-325) demonstrated porcine-type STa toxoids [pSTa(P12F) and pSTa(A13Q)] elicited protective anti-STa antibodies after being fused to a porcine-type LT toxoid [pLT(R192G)]. In this study, we substituted the 8th, 9th, 16th, or the 17th amino acid of a human-type STa (hSTa) and generated 28 modified STa peptides. We tested each STa peptide for toxicity and structure integrity, and found nearly all modified STa proteins showed structure alteration and toxicity reduction. Based on structure similarity and toxic activity, three modified STa peptides: STa(E8A), STa(T16Q) and STa(G17S), were selected to construct LT_192_-STa_-toxoid_ fusions. Constructed fusions were used to immunize mice, and immunized mice developed anti-STa antibodies. Results from this study provide useful information in developing toxoid vaccines against ETEC diarrhea.

**Keywords****:** STa toxin; ETEC; toxoid; LT-STa toxoid fusion; vaccine; diarrhea

**Supplementary Table 1**

Human *est*A gene (STa): 5’ - atg aat agt agc aat tac tgc tgt gaa ttg tgt tgt aat cct gct tgt acc ggg tgc tat taa -3’

Met N S S N Y C C E L C C N P A C T G C Y

**Primers to clone hSTa gene into pUC19:**

hSTapUCHindIII-F: 5’-GCG CAA AGC TTC TGA TTT TGA T-‘3;

hSTapUCBamHI-R: 5’-AGC CAC GGC GGA TCC AAA TAT AAA GGG –‘3.

**Primers for STa toxoids:**

hSTaE8P-F: 5’-AAT TAC TGC TGT CCA TTG TGT TGT –‘3; hSTaE8P-R: 5’-ATT ACA ACA CAA TGG ACA GCA GTA-‘3

hSTaL9V-F: 5’-TAC TGC TGT GAA GTG TGT TGT AAT-‘3; hSTaL9V-R: 5’-TGG ATT ACA ACA CAC TTC ACA GCA -‘3

hSTaL9I-F: 5’-TAC TGC TGT GAA ATC TGT TGT AAT -‘3; hSTaL9I-R: 5’-TGG ATT ACA ACA GAT TTC ACA GCA –‘3

hSTaT16A-F: 5’-CCT GCT TGT GCC GGG TGC TAT TAA-‘3; hSTaT16A-R: 5’-TTA ATA GCA CCC GGC ACA AGC AGG-‘3

hSTaT16P-F: 5’- CCT GCT TGT CCA GGG TGC TAT TAA-‘3; hSTaT16P-R: 5’-TTA ATA GCA CCC TGG ACA AGC AGG ‘-3

hSTaG17A-F: 5’-CCT GCT TGT ACC GCG TGC TAT TAA-‘3; hSTaG17A-R: 5’-TTA ATA GCA CGC GGT ACA AGC AGG-’3

hSTaG17K-F: 5’-CCT GCT TGT ACC AAG TGC TAT TAA -‘3; hSTaG17K-R: 5’-TTA ATA GCA CTT GGT ACA AGC AGG -‘3

hSTaE8A-F: 5’-AAT TAC TGC TGT GCA TTG TGT TGT –‘3; hSTaE8A-R: 5’-ATT ACA ACA CAA TGC ACA GCA GTA-‘3

hSTaE8F-F: 5’-AAT TAC TGC TGT TTC TTG TGT TGT –‘3; hSTaE8F-R: 5’-ATT ACA ACA CAA GAA ACA GCA GTA-‘3

hSTaE8S-F: 5’-AAT TAC TGC TGT TCA TTG TGT TGT –‘3; hSTaE8S-R: 5’-ATT ACA ACA CAA TGA ACA GCA GTA-‘3

hSTaE8G-F: 5’-AAT TAC TGC TGT GGA TTG TGT TGT –‘3; hSTaE8G-R: 5’-ATT ACA ACA CAA TCC ACA GCA GTA-‘3

hSTaE8R-F: 5’-AAT TAC TGC TGT AGA TTG TGT TGT –‘3; hSTaE8R-R: 5’-ATT ACA ACA CAA TCT ACA GCA GTA-‘3

hSTaE8K-F: 5’-AAT TAC TGC TGT AAG TTG TGT TGT –‘3; hSTaE8K-R: 5’-ATT ACA ACA CAA CTT ACA GCA GTA-‘3

hSTaE8Q-F: 5’-AAT TAC TGC TGT CAA TTG TGT TGT –‘3; hSTaE8Q-R: 5’-ATT ACA ACA CAA TTG ACA GCA GTA-‘3

hSTaL9Q-F: 5’-TAC TGC TGT GAA CAA TGT TGT AAT-‘3; hSTaL9Q-R: 5’-TGG ATT ACA ACA TTG TTC ACA GCA -‘3

hSTaL9K-F: 5’-TAC TGC TGT GAA AAG TGT TGT AAT-‘3; hSTaL9K-R: 5’-TGG ATT ACA ACA CTT TTC ACA GCA -‘3

hSTaL9R-F: 5’-TAC TGC TGT GAA AGA TGT TGT AAT-‘3; hSTaL9R-R: 5’-TGG ATT ACA ACA TCT TTC ACA GCA -‘3

hSTaL9G-F: 5’-TAC TGC TGT GAA GGT TGT TGT AAT-‘3; hSTaL9G-R: 5’-TGG ATT ACA ACA ACC TTC ACA GCA -‘3

hSTaL9S-F: 5’-TAC TGC TGT GAA AGT TGT TGT AAT-‘3; hSTaL9F-R: 5’-TGG ATT ACA ACA ACT TTC ACA GCA -‘3

hSTaT16Q-F: 5’-CCT GCT TGT CAG GGG TGC TAT TAA-‘3; hSTaT16Q-R: 5’-TTA ATA GCA CCC CTG ACA AGC AGG-‘3

hSTaT16K-F: 5’-CCT GCT TGT AAG GGG TGC TAT TAA-‘3; hSTaT16K-R: 5’-TTA ATA GCA CCC CTT ACA AGC AGG-‘3

hSTaT16R-F: 5’-CCT GCT TGT AGG GGG TGC TAT TAA-‘3; hSTaT16R-R: 5’-TTA ATA GCA CCC CCT ACA AGC AGG-‘3

hSTaT16G-F: 5’-CCT GCT TGT GGT GGG TGC TAT TAA-‘3; hSTaT16G-R: 5’-TTA ATA GCA CCC ACC ACA AGC AGG-‘3

hSTaT16S-F: 5’-CCT GCT TGT TCC GGG TGC TAT TAA-‘3; hSTaT16S-R: 5’-TTA ATA GCA CCC GGA ACA AGC AGG-‘3

hSTaG17Q-F: 5’-CCT GCT TGT ACC CAG TGC TAT TAA-‘3; hSTaG17Q-R: 5’-TTA ATA GCA CTG GGT ACA AGC AGG-’3

hSTaG17R-F: 5’-CCT GCT TGT ACC AGG TGC TAT TAA-‘3; hSTaG17R-R: 5’-TTA ATA GCA CCT GGT ACA AGC AGG-’3

hSTaG17S-F: 5’-CCT GCT TGT ACC TCC TGC TAT TAA-‘3; hSTaG17S-R: 5’-TTA ATA GCA GGA GGT ACA AGC AGG-’3

hSTaG17F-F: 5’-CCT GCT TGT ACC TTC TGC TAT TAA-‘3; hSTaG17F-R: 5’-TTA ATA GCA GAA GGT ACA AGC AGG-’3
